# Proteomic definition of a desmoglein linear determinant common to *Pemphigus vulgaris *and *Pemphigus foliaceous*

**DOI:** 10.1186/1479-5876-4-37

**Published:** 2006-08-22

**Authors:** Alberta Lucchese, Abraham Mittelman, Luciana Tessitore, Rosario Serpico, Animesh A Sinha, Darja Kanduc

**Affiliations:** 1 Dept. of Odontostomatology, University of Bari, Italy; 2 Dept. of Medicine, New York Medical College, Valhalla, NY, USA; 3 DISCAFF, University A. Avogadro, Novara, Italy; 4 Institute of Clinical Odontostomatology, 2^nd ^University of Naples, Italy; 5 Division of Dermatology and Cutaneous Sciences, Center for Investigative Dermatology, Michigan State University, East Lansing, MI, USA; 6 Dept. of Biochemistry and Molecular Biology, University of Bari, Italy

## Abstract

**Background:**

A number of autoimmune diseases have been clinically and pathologically characterized. In contrast, target antigens have been identified only in a few cases and, in these few cases, the knowledge of the exact epitopic antigenic sequence is still lacking. Thus the major objective of current work in the autoimmunity field is the identification of the epitopic sequences that are related to autoimmune reactions. Our labs propose that autoantigen peptide epitopes able to evoke humoral (auto)immune response are defined by the sequence similarity to the host proteome. The underlying scientific rationale is that antigen peptides acquire immunoreactivity in the context of their proteomic similarity level. Sequences uniquely owned by a protein will have high potential to evoke an immune reaction, whereas motifs with high proteomic redundancy should be immunogenically silenced by the tolerance phenomenon. The relationship between sequence redundancy and peptide immunoreactivity has been successfully validated in a number of experimental models. Here the hypothesis has been applied to pemphigus diseases and the corresponding desmoglein autoantigens.

**Methods:**

Desmoglein 3 sequence similarity analysis to the human proteome followed by dot-blot/NMR immunoassays were carried out to identify and validate possible epitopic sequences.

**Results:**

Computational analysis led to identifying a linear immunodominant desmoglein-3 epitope highly reactive with the sera from *Pemphigus vulgaris *as well as *Pemphigus foliaceous*. The epitopic peptide corresponded to the amino acid REWVKFAKPCRE sequence, was located in the extreme N-terminal region (residues 49 to 60), and had low redundancy to the human proteome. Sequence alignment showed that human desmoglein 1 and 3 share the REW-KFAK–RE sequence as a common motif with 75% residue identity.

**Conclusion:**

This study 1) validates sequence redundancy to autoproteome as a main factor in shaping desmoglein peptide immunogenicity; 2) offers a molecular mechanicistic basis in analyzing the commonality of autoimmune responses exhibited by the two forms of pemphigus; 3) indicates possible peptide-immunotherapeutical approaches for pemphigus diseases.

## Background

There are over 80 autoimmune diseases known or thought to be autoimmune in nature and, as a group, autoimmune diseases affect approximately 20% of the population. Multiple mechanisms of autoimmunity induction have been proposed including, among the others, molecular mimicry [1], viral epitope delivery [2], generation of neoantigenic epitopes after posttranslational modification [3], unusual TCR-binding properties that permit autoreactive T cells to escape deletion [4], presence of long regions of extreme structural disorder in the autoantigens [5], cellular injury and release of self antigens, which generate immune responses [1]. However, the mechanisms leading to the breakdown of tolerance against enzyme autoantigen molecules remain poorly understood. The identification and comparative analysis of the autoantigenic immunoreactive determinants could elucidate the molecular basis of the autoimmune disease and offer new immunotherapeutical approaches.

Our labs have proposed that sequence similarity to the host proteome may influence peptide immunogenicity [6,7]. According to this hypothesis, only peptide motifs having low similarity to the host proteome have the potential to raise an immune response. Conversely, no immune response can be evoked by antigenic protein fragments that are repeatedly represented in the protein set that form the proteome. In testing this work hypothesis, our current research is focused to the proteomic definition of the epitopic peptide repertoire associated to autoimmune diseases. In practice, based on the assumption that peptide sequences that are scarcely represented in human proteins might provoke autoimmune responses by offering antigenic determinants unknown to (or, better, scarcely encountered by) the immune system, we search for epitopic sequences of human pathogenic autoantigens by, first, selecting for autoantigen fragments not shared with the human proteome and, then, analyzing the immunoreactivity pattern of the not-shared peptides using sera from patients hosting the autoimmune disease. So far, the similarity hypothesis has been validated in a number of different experimental models [6-13].

In this study, we used the pemphigus autoimmune skin diseases and the corresponding desmoglein autoantigens as an experimental model [14]. Indeed, pemphigus provides an exemplar paradigm for studying autoimmunity because of its clear-cut antigenic characterization [14,15]. Desmoglein 3 (Dsg3) and desmoglein 1 (Dsg1) are the main autoantigenic target in *Pemphigus vulgaris *(PV) and *Pemphigus foliaceus *(PF), respectively [16,17]. Moreover, epitope mapping revealed that autoantibodies (AAbs) from PV and PF patients recognize conformational epitopes hosted in the amino terminal ectodomain of Dsg3 and Dsg1 respectively [16,18]. However, although it has been reported that pemphigus sera contain antibodies against continuous epitopes [16,19], there is a general lack of data on the definition and specificity of Dsg linear epitopes. Therefore, given also that the molecular definition of linear Dsg autoepitopes could contribute to clarify unresolved aspects of pemphigus autoimmunity [20-22], we undertook an experimental study to define the linear epitopic sequences in Dsg3 by using the criterion of non-redundancy to human proteome as a search engine for epitopic peptides.

Here we describe how the search for sequences uniquely present in the human Dsg3 protein led to identifying an amino terminal linear Dsg3 motif that immunoreacts with sera from *Pemphigus vulgaris *as well as *Pemphigus foliaceous*.

## Materials and methods

### Computer-assisted analyses

EC1/EC2 (aa 1–212) portion of human Dsg3 sequence (SWISS-PROT, P32926) was analysed for redundancy to human proteome using PIR protein database (141702 sequences) and peptide match program (pir.georgetown.edu/pirwww) [23]. Sequence alignment was conducted by using SIM – Local similarity program (www.expasy.org) [24].

### Sera

Sera were obtained from the outpatient Dermatology Clinic at the Weill-Cornell Medical College, New York, and Medical College of Wisconsin, Milwaukee. The diagnosis of PV and PF was made on the basis of clinical examination and biopsy as well as indirect IF serum titer. Sera from prostate cancer patients were used as controls.

### Peptides

Peptides were synthesized using standard Fmoc (N-(9-fluorenyl) methoxycarbonyl) solid phase peptide synthesis (PeptidoGenic Research & Co., Livermore, CA.; Primm srl, Milan, Italy). Peptide purity (>90%) was controlled by analytical HPLC, and the molecular mass confirmed by fast atomic bombardment mass spectrometry. Peptides used for dotblot immunoassay are: Dsg1_49–60_REWIKFAAACRE, Dsg3_36–44_EEMTMQQAK, Dsg3_49–60_REWVKFAKPCRE, Dsg3_190–204_LNSKIAFKIVSQEPA, Dsg3_373–380_QVINVREG and Dsg3_518–525_NRYTGPYT. The ^15^N-labelled peptides used for NMR spectroscopy immunoanalysis are: Dsg3_49–60_REWVKFAKPCRE, Dsg3_373–380_QVINVREG and Dsg3_518–525_NRYTGPYT peptides (with ^15^N-labelled amino acid residues given underlined). Control recombinant proteins corresponding to the EC domains of Dsg1 and Dsg3 [25] were generously provided by Dr. M.S. Lin, Dept. of Dermatology, Medical College of Wisconsin.

### Immuno-blot assays

Pemphigus serum reactivity against synthetic peptides was tested by immunodotblot assays. Nitrocellulose membrane (0.2 μm pore size, Biorad Laboratories, Milan, Italy) was pretreated with 1% glutaraldehyde. Dsg3 protein (10 μg) or peptides (4 μg) were spotted on the activated membranes and immunoassayed with PV or PF AAbs [8,11,13]. For Western blot assay, Dsg1 and Dsg3 proteins were resolved on sodium dodecylsulfate-10% polyacrylamide gel electrophoresis (SDS-10%PAGE), electroblotted onto PVDF membrane (Biorad Laboratories, Milan), and probed with PV or PF sera.

### NMR spectroscopy

Spectroscopic analyses were carried out on sera pooled and partially purified by precipitation with 40% saturated (NH4)_2_SO_4 _(x 2). The precipitate was dissolved in phosphate-buffered saline (PBS), dialyzed against PBS with several changes for 24 h at 4°C, then aliquoted and stored at -20°C until assay. NMR spectra of the reaction of the synthetic ^15^N-labelled Dsg3_49–60 _REWVKFAKPCRE, Dsg3_373–380_QVINVREG or Dsg3_518–525_NRYTGPYT peptide (with ^15^N-labelled amino acid residues given underlined) with partially purified AAbs from pooled sera of PV or PF or prostate cancer patients as control were recorded at 298°K on a Bruker Avance DRX500WB spectrometer. The spectra were acquired by heteronuclear single quantum correlation (HSQC) experiments as already detailed [8,11,26]. We used chemical shift statistics from the full BioMagResBank database, where the calculated statistics are derived from a total of 559392 chemical shifts (www.bmrb.wisc.edu). Sequence-specific correction factor tabulations were applied to backbone ^1^H and ^15^N resonances [27]. Two-dimensional correlated spectroscopy spectra of peptide-AAbs complex were obtained using peptide:AAb ratio equal to 0.1:30, mg/mg. That is, NMR samples contained either 0.1 mg free Dsg3_49–60_REWVKFAKPCRE (or Dsg3_373–380_QVINVREG or Dsg_518–525_NRYTGPYT) peptide; or 30 mg PV AAbs complexed with 0.1 mg Dsg3_49–60_REWVKFAKPCRE (or Dsg3_373–380_QVINVREG or Dsg3_518–525_NRYTGPYT) peptide; or 30 mg PF AAbs complexed with 0.1 mg Dsg3_49–60_REWVKFAKPCRE (or Dsg3_373–380_QVINVREG or Dsg3_518–525_NRYTGPYT) peptide; or 30 mg control AAbs complexed with 0.1 mg Dsg3_49–60_REWVKFAKPCRE (or Dsg3_373–380_QVINVREG or Dsg3_518–525_NRYTGPYT) peptide, in 0.5 ml aqueous solution H_2_O/D_2_O (9:1, v/v).

## Results

### Searching EC1/EC2 Dsg 3 for sequences non-redundant to the human proteome

Since the Dsg antigenic portions recognized by AAbs from PV patients mainly map to the NH^2^-terminal adhesive domain9 [18,28], this preliminary study started by focusing on the extracellular (EC) portion of human Dsg3. The EC_1_/EC_2 _domain of Dsg3, spanning from amino acid 1 to 212, was searched for potential epitopic linear sequences with low redundancy to the self-proteome using the PIR protein database. Matching analysis to the human proteome was performed using pentamer probes, being 5 to 6 amino acids the minimal immunoreactive peptide length [29-31] Redundancy of a peptide sequence is defined here by the number of identical pentamers in common between the analyzed autoantigen and the human proteome.

**Figure 1:**
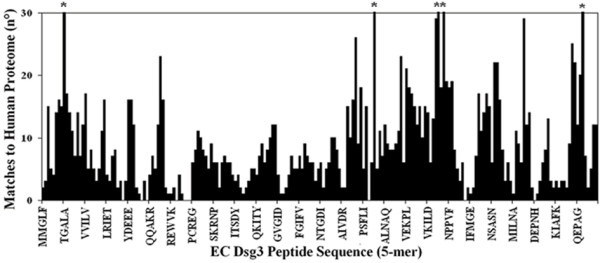
Redundancy profile of the NH_2 _terminal portion of Dsg3 to human proteome. The EC1/EC2 Dsg3 sequence aa1–212. was scanned for perfect matches to human protein sequences by using pentamers offset by one residue. Asterisk: value > 30.

**Figure 2:**
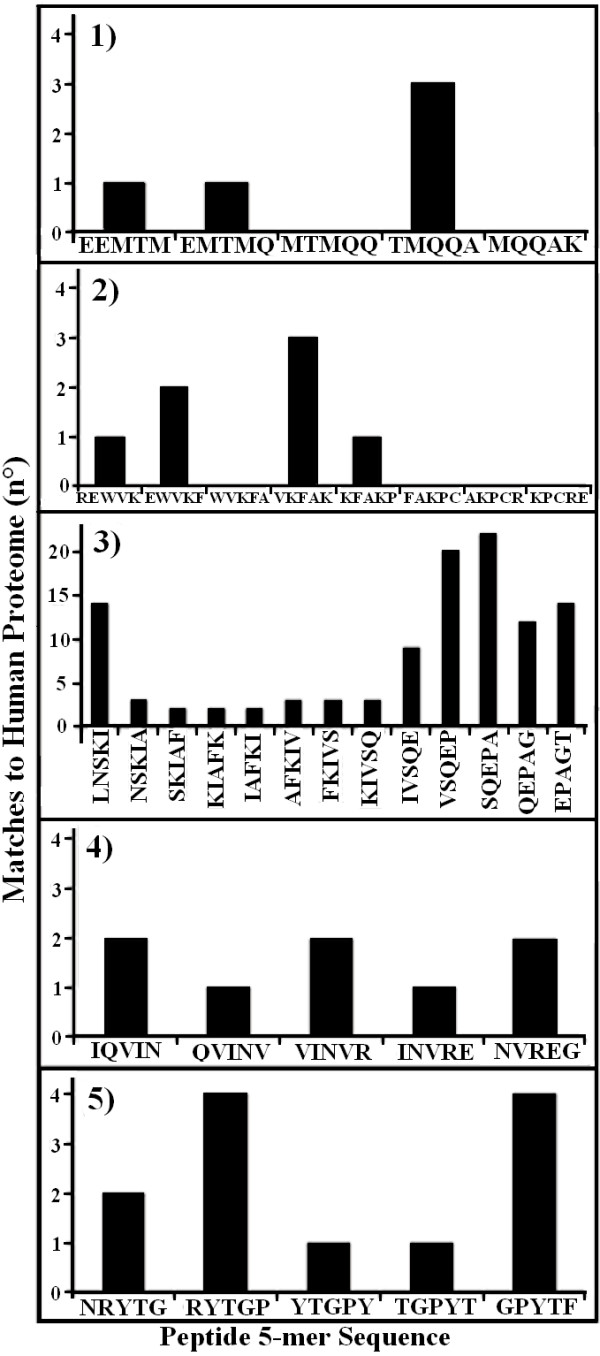
Redundancy scanning on the EC Dsg3 peptide sequences utilized in dot immunoassay analyses with human PV and PF sera. Matching analysis to the human proteome was performed using 5-mer peptide probes. **Peptide: **1) Dsg3_36–44_EEMTMQQAK; 2) Dsg3_49–60 _REWVKFAKPCRE; 3) Dsg3_190–204_LNSKIAFKIVSQEPA; 4) Dsg3_373–380_QVINVREG; 5) Dsg3_518–525_NRYTGPYT.

The Dsg3 sequence was dissected into 5-mer motifs that were used as probes to scan the entire human proteome databank. The Dsg3 pentamers were offset by one residue, i.e. overlapped by four amino acids: MMGLF, MGLFP, GLFPR, LFPRT, FPRTT, etc. The computational analysis produced the histogram reported in Fig. 1. It can be seen that the majority of EC Dsg3 pentamers have a number of perfect match hits in the human proteome, by being shared by a number of other human proteins. Only a few peptide stretches are peculiarly owned by the EC1/EC2 of PV Dsg3 antigen. According to the redundancy hypothesis advanced by our labs [6-13], the non-redundant peptide motifs, i.e. the sequences having a low number of matches (or none at all) in common with the human proteome, might be potentially immunogenic by offering possible epitopic linear determinants unknown to (or rarely seen by) the immune system. In this context, two peptides corresponding to Dsg3_36–44_EEMTMQQAK and Dsg3_49–60_REWVKFAKPCRE sequences appeared of particular interest, since these fragments present lowest similarity to the human proteins and, additionally, host zero match pentamers. Therefore, the corresponding peptides were synthesized to be used in dot immunoassay with AAbs from PV or PF patient sera. A peptide corresponding to Dsg3_190–204_LNSKIAFKIVSQEPA sequence was also synthesized since: i) susceptibility to PV is strongly linked to DRB1*0402 serotype; ii) the Dsg3_190–204_LNSKIAFKIVSQEPA peptide has been reported to bind DRB1*0402; iii) moreover, this peptide appears to be a target for autoreactive T cells in PV patients with active disease [32]. In addition, two Dsg3 synthetic peptides, Dsg3_373–380_QVINVREG and Dsg3_518–525_NRYTGPYT, were used as controls having low redundancy to the human proteome (8 and 12 total matches, respectively, in common with the human proteome), but no pentamers with zero similarity to the human proteome. The similarity profile scanning of the Dsg3 synthetic peptides used in dot-immunoassay analyses with sera from PV and PF patients is illustrated in Fig. 2.

### Immunoreactivity of non-redundant EC1/EC2 Dsg3 peptides

The synthetic peptides, selected as above described and illustrated in Fig. 2, were bound to activated nitrocellulose membrane and tested as potential antigenic epitopes against serum from PV patients in dot immunoassays. Because of reports of anti-Dsg immune responses in healthy individuals [33-35], sera from prostate cancer patients were used as controls. Sera from PF patients were used as additional controls.

**Figure 3:**
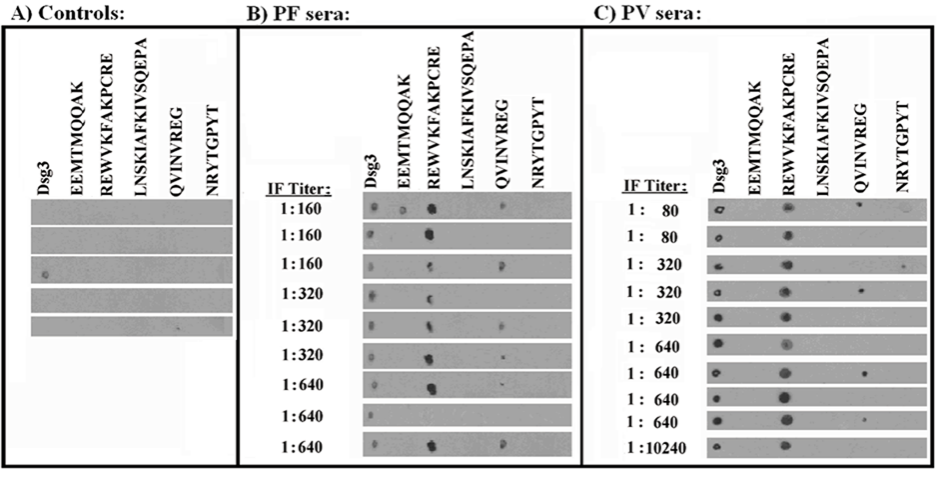
Immunoreactivity of PV, PF or prostate cancer human sera towards EC Dsg3 peptides. **Sera: **A) control sera from prostate cancer patients; B) *Pemphigus foliaceous *sera; C) *Pemphigus vulgaris *sera. Immunoreactivity of each serum was assayed by dot-blot as described under Methods. Indirect IF titer is reported for each PF and PV serum. **Antigens: **Dsg3, recombinant EC Dsg 3 protein; peptides with low redundancy to the human proteins: Dsg3_36–44_EEMTMQQAK and Dsg3_49–60 _REWVKFAKPCRE; peptide with high affinity to DRB*0402: Dsg3_190–204_LNSKIAFKIVSQEPA; control peptides: Dsg3_373–380_QVINVREG and Dsg3_518–52_5NRYTGPYT.

Fig. 3 shows that the non-redundant Dsg3_49–60_REWVKFAKPCRE peptide was immunoreactive with all sera from PV. The Dsg3_373–380_QVINVREG sequence showed some faint reactivity. The DRB1*0402 binding and T cell epitope Dsg3_190–204_LNSKIAFKIVSQEPA peptide [32] did not react with any of the human sera used in this study. Interestingly Fig. 3 illustrates that the non-redundant Dsg3_49–60_REWVKFAKPCRE peptide was immunoreactive with AAbs from PF too.

### NMR probing of Dsg3_49–60_REWVKFAKPCRE peptide immunoreactivity

**Figure 4:**
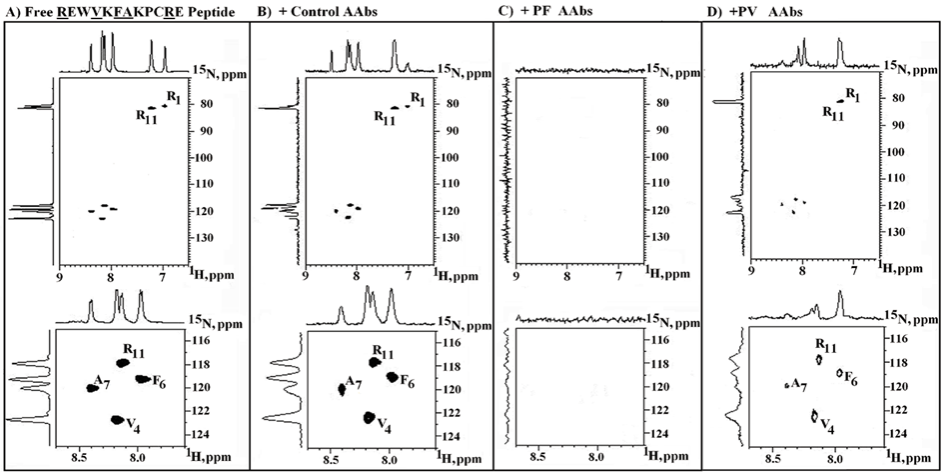
^1^H-^15^N NMR HSQC spectra and relative 1-D contour plots of the low- redundancy Dsg3_49–60_REWVKFAKPCRE peptide ^15^N-labelled at residues 1, 4, 6, 7 and 11. A) free peptide in solution; plus AAbs from B) prostate cancer, C) PF, or D) PV patients. **Upper panels: **portions of the HSQC spectra showing resonances from residues 1, 4, 6, 7 and 11. **Lower panels: **expanded region of the HSQC spectra showing resonances from the residues 4, 6, 7 and 11, by being 1-Arg at terminal position. ^15^N-labelled residues are given underlined.

**Figure 5:**
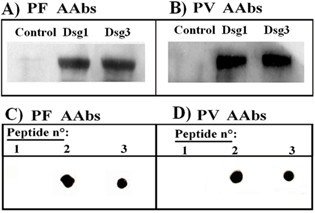
Cross-reactivity of human recombinant EC Dsg1 or EC Dsg3 proteins with PF and PV sera. **Panels A and B**: Western blot analysis of PF or PV serum immunoreactivity towards human recombinant EC Dsg1 or EC Dsg3 protein. Control: phosphorylase was used as a control protein. **Panels C and D: **Dot blot analysis of PF or PV serum immunoreactivity towards peptide: 1) Dsg3_190–204_LNSKIAFKIVSQEPA, 2) Dsg3_49–60 _REWVKFAKPCRE, and 3) Dsg1_49–60_REWIKFAAACRE.

False positives as well as false negatives are a constant feature of immunoassays. Consequently, we controlled the dot-blot results reported in Fig. 3 by NMR spectroscopy to irrefutably demonstrate the immunoreactivity of Dsg3_49–60_REWVKFAKPCRE peptide with both PV and PF AAbs. NMR spectroscopy measures the antigen-antibody binding reaction by monitoring nuclear chemical shifts, i.e. the motional freedom of nuclei. However, application of NMR technology to the analysis of the antigen-antibody interaction is not unproblematic, since the high number of amino acid residues complicates the assignment of specific chemical shift signals. Therefore, we enhanced the NMR detection limit by measuring one-bond proton-nitrogen shift correlations with two-dimensional (2-D) phase-sensitive pulsed field gradient HSQC experiments [26]. In addition, synthetic peptides containing ^15^N-labelled amino acids were used in order to obtain greater intensities and unequivocal resolution. Specifically, the non-redundant Dsg3_49–60_REWVKFAKPCRE, Dsg3_373–380_QVINVREG, and Dsg3_518–525_NRYTGPYT peptides (with ^15^N-labelled amino acid residues given underlined) were synthesized and chemical shift changes following AAb addition were monitored in two-dimensional correlated spectroscopy spectra as reported in Fig. 4, 5, and 6, respectively. Each spot in the figures is an NMR signal representing the ^1^H-^15^N one-bond coupling of the labelled amino acid residues in the peptide.

Table 1 lists the numerical values of the theoretical and experimental chemical shifts of the ^15^N-labelled amino acid residues in the Dsg3_49–60_REWVKFAKPCRE peptide. Theoretical values were calculated by using chemical shift statistics from BioMagResBank (www.bmrb.wisc.edu) plus sequence-dependent factors to correct deviations due to local sequence effects [27]. Experimental values derive from the resonance spectrum of the reactions illustrated in Fig. 4, and indicate the chemical shift of the labelled amino acid residues in the free peptides in solution vs. those obtained following AAb addition to the peptide solution.

**Table 1:** Chemical shifts of ^15^N-labelled residues in Dsg_49-60_REWVKFAKPCRE peptide, and changes on control, PV or PF Aab addition

^15^N-residue^1^	Chemical Shift Values:									
	Theoretical^2^:		Experimental^3^:							

	Free peptide		Free peptide		Control Aabs		+PV Aabs		+PF Aabs	

	^1^H^N^	^15^N	^1^H^N^	^15^N	^1^H^N^	^15^N	^1^H^N^	^15^N	^1^H^N^	^15^N

1-Arg^4^	6.78	77.6	6.95	80.6	7.02	80.7	7.24	81.2	-	-
4-Val	8.65	120.8	8.18	122.7	8.18	122.5	8.16	122.5	-	-
6-Phe	8.42	121.7	7.97	119.3	7.97	119.0	7.96	119.0	-	-
7-Ala	8.29	122.3	8.40	120.0	8.40	120.0	8.38	119.8	-	-
11-Arg^4^	6.78	77.6	7.22	81.4	7.27	81.6	7.20	81.6	-	-
	8.14	119.6	8.14	117.9	8.13	117.7	8.12	117.9	-	-

^115^N-amino acid position in the peptide. ^2 ^Theoretical chemical shift values were derived from bmrb.wisc.edu, and corrected for deviations due to local sequence effects [27]. ^3 ^Experimental values from resonance spectra reported in Fig. 4. ^4 ^Average value of chemical shifts relative to the H and N atoms of η amino residues in arginine. The chemical shifts relative to the H and N atoms of α amino residue are undetectable in 1-Arg because of the NH_2 _terminal position.

Specifically, Fig. 4 shows the reaction between human AAbs and the immunoreactive Dsg3_49–60_REWVKFAKPCRE peptide. Fig. 4A reports the Arg, Val, Phe, and Ala selective ^1^H-^15^N correlations of the free peptide in aqueous solution, with the upper part of Fig. 4A displaying the Arg cross-peak signals due to the H and N atoms of amino η residues. On the whole, Fig. 4A clearly? shows that all of the expected signals are present and in basic agreement with the theoretical data relative to the ^15^N-labelled amino acids (see chemical shift values in Table 1). The addition of AAbs from control cancer sera did not alter the REWVKFAKPCRE resonance signals, so that the spectrum of Fig. 4B could be assigned by reference to the control spectrum of the free peptide reported in Fig. 4A, both qualitatively and quantitatively. On the contrary, a complete signal deletion was provoked by the addition of AAbs from PF sera as illustrated in Fig.4C, upper and lower parts. Practically, Fig. 4C demonstrates that the PF AAbs were able to specifically neutralize and quantitatively precipitate the low similarity Dsg3_49–60_REWVKFAKPCRE peptide. In the presence of PV AAbs, well resolved resonances for ^15^N-labelled epitopic REWVKFAKPCRE peptide residues were detectable (Fig. 4D, upper and lower panels), but the signal intensity was much weaker when compared to the control spectra reported in Fig. 4, panels A and B. That indicates that only a part of the ^15^N-labelled peptide had been complexed and buried by PV AAbs. No precipitation reaction was observed by using the control ^15^N-labelled Dsg3_373–380_QVINVREG and Dsg3_518–525_NRYTGPYT peptides (not shown).

### Dsg1 and Dsg3 cross-react with PF and PV sera: sharing of the 75% consensus motif REW-KFAK–RE

It is *res iudicata *in pemphigus pathologies that Dsg3 is the autoantigen of the vulgaris form, whereas Dsg1 is the autoantigen of the foliaceous form [14,15,17-19,33]. In agreement with this statement, in the experiments illustrated in Figs. 3 and 4, PF AAbs were initially used as additional controls. On the other hand, the peculiar data emerging from Figs. 3 and 4 is the highly specific reaction between the PF AAbs preparation and the peptide from PV-associated Dsg3 autoantigen. Indeed, given the caveat that the extent of the ^15^N-labelled peptide reaction monitored in the NMR spectroscopy analyses depends on the AAb titer of each of the pooled sera, the NMR spectra reported in Fig. 4 indicated that the reaction extent with AAbs from PF sera was even higher than that monitored with AAbs from PV sera. The finding was of interest also in the light of reports indicating a common desmoglein background in PV and PF, and coexistence of the two pemphigus forms in the same patient [36,37].

To better understand and define the cross-reactivity between the PF AAbs and the PV associated Dsg3 peptide, we analyzed Dsg1 and Dsg3 protein immunoreactivity towards AAbs from PV and PF by Western blot analysis. Fig. 5, panels A and B, show that: i) both PV and PF AAbs recognize a linear desmoglein determinant; ii) the linear desmoglein determinant is common to Dsg1 and Dsg3, so confirming the NMR spectroscopic immunoreaction reported in Fig. 4C.

The cross reaction between the PF AAbs and the PV associated Dsg3 peptide was further investigated by Dsg1/Dsg3 sequence alignment. The sequence analysis demonstrates 75% identity in the fragment spanning aa 49 to 60:



with REW-KFAK–RE as a common consensus motif, and suggests that the shared amino acids of the consensus motif may represent the epitopic residues recognized by both PV and PF sera, so providing a theoretical molecular framework to the data reported in Figs. 3, 4 and 5. Final experimental confirmation was obtained in immunodotblot assays by using the synthetic Dsg1_49–60_REWIKFAAACRE peptide with PV and PF sera. Fig. 5, panel C, illustrates that PF AAbs recognize both Dsg1_49–60_REWIKFAAACRE and Dsg3_49–60 _REWVKFAKPCRE peptides. Likewise, also PV AAbs recognize Dsg1_49–60_REWIKFAAACRE as well as Dsg3_49–60_REWVKFAKPCRE peptide (Fig. 5, panel D).

## Discussion

Using computational biology and proteomics, and applying sequence uniqueness as a search criterium, we have characterized a linear low-redundant Dsg3 segment which is immunorecognized by sera from PV patients as well as PF patients. These data appear of scientific, clinical, and therapeutical interest.

Scientifically, the present study demonstrates that analytical dissection of the human proteome allows to comparatively analyze the molecular basis of complex multi-faceted diseases such as the pemphigus, and precisely identify autoantigen portions involved in immune responses. As a matter of fact, the methodology used in this study was extremely effective for individuating one new desmoglein epitope. Sets of 40 synthetic peptides, each 15 residues in length and overlapping by ten amino acid residues, should had been screened to scan the EC1/EC2 Dsg3 sequence by using canonical peptide-mapping analysis. In our approach identification of an immunodominant linear desmoglein epitope involved only 6 synthetic peptides. The application of the proteomic approach to find unique sequences led to rapid, effective and inexpensive epitope identification when compared to other methods,

Clinically, we note that although other investigators have analyzed desmogleins for patterns of continuous immunoreactive peptides [19,38,39], this is the first time, as far as we know, that a linear determinant on Dsg3 is identified as a precise amino acid sequence immunoreactive with patients' sera. Likewise, this is the first time that a common link between PV and PF is presented in terms of a common Dsg3/Dsg1 immunoreactive motif, i.e. the consensus REW-KFAK–RE sequence. Given the caveat that immunogenicity and pathogenicity are not synonymous and further studies are necessary in order to assess the (patho)physiological significance of the present data, undoubtedly these experimental results might be of help in defining the autoantibody response of pemphigus diseases. Indeed, pemphigus is a group of diseases of the skin and mucous membranes that 1) include different forms and variants, 2) are characterized by complex autoantibody profiles, and 3) present blistering process as a minimum common clinical denominator [40-42]. Consequently, a major goal in understanding pemphigus autoimmunity is the identification of critical epitopes along the antigen portions involved in the calcium-dependent cell-cell adhesion process. In this context, the data presented in this work, describing the NH2 terminal calcium-adhesive Dsg3_49–60_REWVKFAKPCRE immunoreactive peptide, might represent a prelude to an exact definition of the entire Dsg linear epitope pattern. Indeed, the individuation of the consensus motif as a common sequence recognized by PF and PV sera is an experimental point that might help in the fine definition of the AAb profile in pemphigo diseases. Moreover, it has to be mentioned that also the monoclonal humoral response to the ectodomain of human Dsg3 targets within the Dsg3_49–60 _REWVKFAKPCRE sequence [13]. This is an additional reason for further studying Dsg3_49–60 _sequence in the context of the induction of bullous skin disease resembling PV through passive transfer of PV AAbs [43].

Therapeutically, the usage of proteomic sequence uniqueness as a guiding principle in identifying epitopic sequences along autoantigenic proteins offers the possibility of effective peptide-immunotherapies in autoimmune diseases. The peptide-immunotherapy approach is currently being explored with encouraging results in many autoimmune diseases [44-46]. Indeed, short peptide fragments might be used to selectively block and neutralize autoantibodies, once the effective epitopic sequences have been determined and the definition between immunogenic and pathogenic epitopes is clearly drawn. Moreover, the precise identification of amino acid sequences uniquely present in the autoantigen of interest would also eliminate the side effects deriving from possible cross-reactions. In this context, the proteomics-defined REWVKFAKPCRE sequence represents a suitable candidate for a possible peptide-immunotherapy in pemphigus diseases.

## Abbreviations

Dsg, desmoglein; EC, extracellular; PV, *Pemphigus vulgaris; *PF, *Pemphigus foliaceous; *HSQC, heteronuclear single quantum correlation; AAbs, autoantibodies.

## Authors' contributions

All Authors have made substantive intellectual contributions to the study conception and design as well as in drafting, modifying and revising the manuscript. In addition, AL has been involved in immunoassays; AAS in sera collection and analysis; DK in NMR studies.
